# Risk Predictors of 3-Month and 1-Year Outcomes in Heart Failure Patients with Prior Ischemic Stroke

**DOI:** 10.3390/jcm11195922

**Published:** 2022-10-07

**Authors:** Ding Li, Yu Wang, Feng Ze, Xu Zhou, Xue-Bin Li

**Affiliations:** 1 Department of Cardiology, Peking University People’s Hospital, Beijing 100044, China; 2 Department of Cardiology, Chinese-Japanese Friendship Hospital, Beijing 100013, China

**Keywords:** heart failure, ischemic stroke, readmissions, death

## Abstract

*Background:* Despite available therapy, mortality, and readmission rates within 60–90 days of discharge for patients hospitalized with heart failure (HF) are higher compared to the 1-year rates. This study sought to identify the risk factors of the combined endpoint of all-cause readmission or death among HF patients. *Methods:* Patients with a diagnosis of HF aged 65 or older were included in this prospective observational cohort study. The outcomes were estimated within 3-months and 1 year of discharge. Risk modeling was performed using a multivariable Cox regression analysis of HF patients older than 65 who had experienced ischemic stroke. *Results:* A total of 951 HF patients enrolled, of whom 340 (35.8%) had suffered a prior ischemic stroke. Significant predictors of increased 3-month all-cause readmission or death included DBP (*p* = 0.045); serum albumin (*p* = 0.025), TSH (*p* = 0.017); and discharge without ACE-inhibitor/ARB/ARNI (*p* = 0.025), β-blockers (*p* = 0.029), and antiplatelet drugs (*p* = 0.005). Heart rate (*p* = 0.040), laboratory parameters—including serum albumin (*p* = 0.003), CRP *p* = 0.028), and FT4 (*p* = 0.018)—and discharge without β-blockers (*p* = 0.003), were significant predictors of increased 1-year all-cause readmission and death. *Conclusions:* Without β-blockers, lower serum albumin and abnormal thyroid function increase the risks of readmission and death in elderly HF patients who have had an ischemic stroke by 3 months and 1 year after discharge. The other factors, such as being without ACEI/ARB and a high heart rate, only increase risks before 3 months or 1 year, not both.

## 1. Introduction

Heart failure (HF) is a rapidly growing public health issue with an estimated prevalence of >37.7 million individuals globally [1]. In China, 1.3% (estimated 13.7 million) of the Chinese adult population aged ≥35 years has experienced HF [2]. 

After an episode of worsening chronic heart failure, rates of readmission to the hospital and death are high, especially in the first few months following said episode [3]. The early post-discharge period immediately following hospitalization carries a particularly high risk of poor clinical outcomes and is known as the “vulnerable phase” [4]. Based on this concept, increased attention should be given to transitions of care, pre-discharge quality measures and early post-discharge clinic visits and monitoring. An urgent need exists for novel approaches to improve early post-discharge outcomes.

Stroke is the most common cause of disability and a major cause of death in developing countries, especially China [5]. A strong interaction between HF and ischemic stroke is well established; there is great deal of evidence that HF itself increases the risk of a thromboembolism, which can lead to a stroke independently of atrial fibrillation, which in turn substantially contributes to morbidity and mortality in HF patients, and several pathophysiologic mechanisms have been described [6]. It has been proved that a prior ischemic stroke is a potent and persistent risk predictor of death and readmission among patients with HF after accounting for clinical characteristics [7]. However, evidence of risk factors on all-cause readmission or death in HF patients with prior ischemic stroke particular is relatively limited.

Thus, estimating the risk factors of HF patients with prior ischemic stroke in different phases is an important step towards reducing hospital readmissions rates in subgroups of HF patients. Therefore, our study aimed mainly to illuminate risk factors for all-cause readmission or death in HF patients with prior ischemic stroke in different phases. An understanding of this could have important implications for future prevention strategies for these certain patients with HF.

## 2. Materials and Methods

### 2.1. Study Population

**Figure 1:**
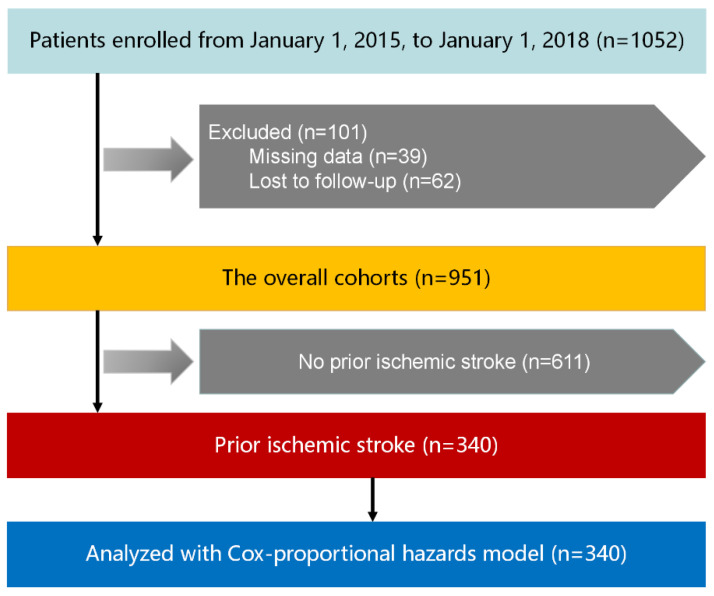
Selection process of patients.

This was a prospective observational cohort study of patients discharged from Peking University People’s Hospital between 1 January 2015 and 1 January 2018. Patients older than 65 years admitted to the department with a diagnosis of HF were included in this study. Patients with HF were identified based on the clinical practice guidelines by the Chinese Society of Cardiology [8], whose diagnostic criteria are similar to the recommendations of the European Society of Cardiology [9]. A patient with not-well-controlled cancer and another active disease was excluded from this study. A total of 951 cases met our inclusion criteria. The ethics committee of Peking University People’s Hospital approved the overall protocol. Figure 1 shows the patient selection process.

### 2.2. Diagnosis of Prior Ischemic Stroke

Patients who had suffered an ischemic stroke were clinically assessed according to the guidelines by the American Heart Association/American Stroke Association with minor modifications. The assessment was briefly based on: (1) neurological deficits lasting more than one month because of ischemic lesions or transient ischemic attacks and (2) an imaging examination, which showed an infarction lesion related to the past clinical findings. In 340 of these patients, prior ischemic stroke was diagnosed [10].

### 2.3. Data Collection 

The variables collected in the present study included demographics (age, sex, and history of smoking), echocardiographic information, biochemical data (albumin, brain natriuretic peptide, blood urea nitrogen, etc.), comorbidities at admission (cardiac and noncardiac comorbidities), and medications (angiotensin-converting enzyme inhibitors (ACEIs) or angiotensin receptor blockers (ARBs), ARNI, β-blockers, mineralocorticoid receptor antagonists (MRAs), calcium channel blockers, statins, diuretic, nitrates, digoxin, antiplatelet drugs, and anticoagulants) at admission and discharge. 

### 2.4. Study Outcomes

We examined the following outcomes: the combined endpoint of all-cause readmission or death, all-cause death, all-cause readmission, and HF readmission. We performed follow-up interviews regularly, and all events of death or readmission by 3 months and by 1 year were documented on the China Heart Failure Center Reporting Platform (http://data.chinahfc.org, accessed on 1 October 2022). Only the first readmission occurring at any time after the index admission and throughout the follow-up period of this study was considered to be the qualifying readmission. The definition of HF readmission is readmission due to recurrent HF.

### 2.5. Statistical Analysis

Normally distributed continuous variables are expressed as mean ± standard deviation; non-normally distributed continuous variables are expressed as median and interquartile range, and categorical variables are expressed as *n* (%). Pearson chi-square and Wilcoxon rank-sum tests were used for the comparisons of baseline characteristics between patients with and without all-cause readmission or death, as appropriate. Univariate variables with *p* values of equal to or less than 0.1 were included in the multivariate Cox regression analysis. The cumulative risks for outcomes were estimated by the Kaplan–Meier survival curves and compared using the COX regression analysis. The hazard ratio (HR) and 95% confidence intervals (CI) express the risks of coronary and cardiovascular events of the cohort with all-cause readmission or death compared to the cohort without all-cause readmission or death. Two-sided tests with *p* < 0.05 were considered statistically significant. Statistical analysis was conducted using SPSS for Windows (version 25.0, SPSS Inc., Chicago, IL, USA).

## 3. Results 

### 3.1. Baseline Characteristics in the Entire Cohort

All 951 patients had a mean age of 76.95 ± 6.52, and 46.9% were female. In total, 340 (35.8%) patients had suffered a prior ischemic stroke. These had a greater burden of comorbidities and underwent less guideline-directed medical therapy than those patients without prior ischemic stroke (Table 1). 

### 3.2. Month Outcomes

**Figure 2:**
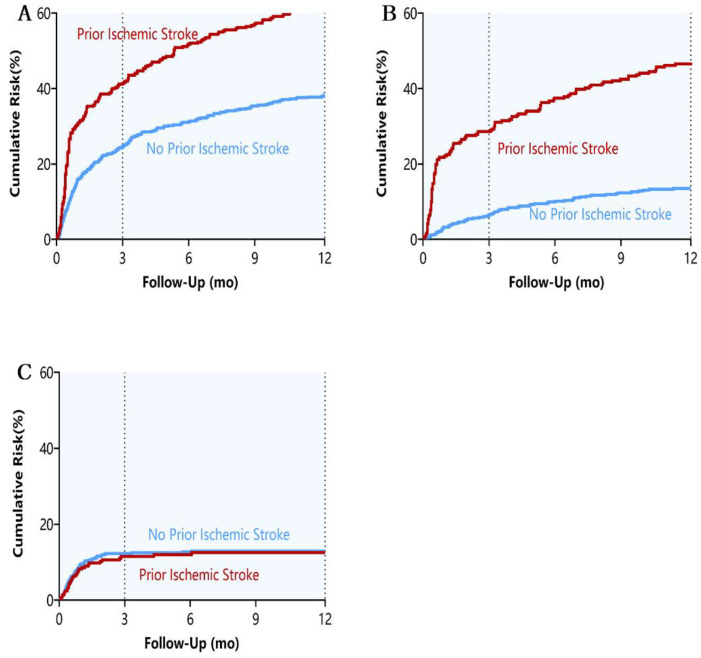
(**A**) All-cause death or readmission; (**B**) Non-HF readmission; (**C**) All-cause death.

Prior ischemic stroke was associated with significantly higher risks of the combined endpoint of all-cause death or readmission (HR: 1.75; 95% CI: 1.39 to 2.19; *p* < 0.001) and all-cause readmission (HR: 2.33; 95% CI: 1.76 to 3.06; *p* < 0.001) (Table 2; Figure 2). These differences were mainly due to non-HF readmission (HR: 4.48; 95% CI: 3.09 to 6.51; *p* < 0.001), not HF readmission or all-cause death. 

### 3.3. Year Outcomes

Prior ischemic stroke was also associated with higher risks of the combined endpoint of all-cause death or readmission, all-cause readmission, and non-HF readmission at 1 year (Table 2; Figure 2), similarly to the 3-month outcomes. 

### 3.4. Multivariable Analysis of Factors for 3-Month All-Cause Readmission or Death in HF Patients with Prior Ischemic Stroke

The results of the multivariable Cox regression analysis for 3-month outcomes are shown in Table 3. Diastolic blood pressure (DBP) (HR: 0.99; 95% CI: 0.97 to 1.00; *p* = 0.045), laboratory parameters—including serum albumin (HR: 0.95; 95% CI: 0.90 to 0.99; *p* = 0.025) and thyroid-stimulating hormone (TSH) (HR: 1.04; 95% CI: 1.01 to 1.08; *p* = 0.017)—and medication, including discharge without ACE-inhibitor/ARB/ARNI (HR: 0.59; 95% CI: 0.37 to 0.93; *p* = 0.025), beta-blockers (HR: 0.62; 95% CI: 0.40 to 0.95; *p* = 0.029), and antiplatelet drugs (HR: 0.51; 95% CI: 0.32 to 0.82; *p* = 0.005) were significant predictors of increased all-cause readmission or death.

### 3.5. Multivariable Analysis of Factors for 1-Year All-Cause Readmission or Death in HF Patients with Prior Ischemic Stroke

The results of the multivariable Cox regression analysis for 1-year outcomes are shown in Table 4. Heart rate (HR: 0.99; 95% CI: 0.97 to 1.00; *p* = 0.040), laboratory parameters—including serum albumin (HR: 0.93; 95% CI: 0.87 to 0.97; *p* = 0.003), C-reactive protein (HR: 1.01; 95% CI: 1.00 to 1.02; *p* = 0.028), and free thyroxin (HR: 1.08; 95% CI: 1.01 to 1.15; *p* = 0.018)—and discharge without beta-blockers (HR: 0.53; 95% CI: 0.35 to 0.80; *p* = 0.003) were significant predictors of increased all-cause readmission or death.

## 4. Discussion

The findings from the present study demonstrated that prior ischemic stroke in patients with HF was associated with significantly higher risks of poor outcomes, which is in agreement with previous analyses. Here, we have reported several predictors of all-cause readmission by 3 months and by 1 year for HF patients with prior ischemic stroke. The present analysis was the first to evaluate the risk factors of early vs. late readmission after an initial hospitalization for HF patients with prior ischemic. These findings complement and extend knowledge about the prognostic value of ischemic stroke among patients with HF.

Patients who are discharged after HF-related hospitalization enter what is called the “vulnerable phase” [11]. Although it is not clearly defined, the vulnerable phase includes the period immediately after discharge until 2–3 months later and is marked by high rates of mortality and readmission [11]. Current guidelines and financial penalties place a strong emphasis on minimizing vulnerable-phase rehospitalization or death, with a particular focus on HF in a broad sense, which does not consider certain clinical phenotypes of HF. The present findings show that HF patients have increased risks of early and late all-cause readmission if they have a history of prior ischemic stroke within the past year. Additionally, the length of the vulnerable phase in HF patients with prior ischemic stroke is significantly longer than that of normal HF patients. The present findings, combined with existing work, support the idea that we need to put emphasis on early follow-up appointments and increased preventive efforts during the discharge phase for HF patients with prior ischemic stroke.

In order to explore the risk factors of readmission by 3 months and by 1 year in HF patients with prior ischemic stroke, we evaluated clinical profiles, cardiac and noncardiac comorbidities, and medications. Finally, we established two multivariate Cox regression analysis models to determine independent prognosticators of readmission in the two phases. Our study has several strengths, one of which is that it highlights the influences of certain variables on all-cause readmission or death, which is attributed to the fact that we considered distinct clinical profiles in different phases. Many of these factors differ substantially in absolute terms, and therefore might be used for rapid risk stratification in routine clinical practice. 

### 4.1. Antiplatelet Therapy 

Actually, among patients with HF who were in sinus rhythm, there was no significant overall difference in the primary outcomes between treatment with warfarin and treatment with aspirin, which was proved by several trials. However, one noteworthy finding of this study was that the use of antiplatelets during the vulnerable phase was associated with a decreased risk of poor outcomes. Several mechanisms are thought to be responsible for this. One of the most important reasons for this is that, for the large subset of patients with poor early-post-discharge cardiovascular outcomes, particularly HF rehospitalization, the underlying pathophysiology is typically related to the short-term worsening of hemodynamics, primarily increasing left ventricular filling pressure [3,12,13]. These changes may contribute to thromboembolism according to the increased aggregation of thrombocytes, reduced fibrinolysis, endothelial dysfunction, inflammatory activation, and malfunctioning of cerebral autoregulation [6]. Thus, targeting of platelets in therapy in the vulnerable phase may reduce the risk of secondary ischemic stroke. Second, an ischemic stroke may lead to dysphagia-related pulmonary aspiration, physical function impairment, or cognitive impairment [14,15]; this is the reason for increased morbidity and mortality.

### 4.2. Thyroid Disease

Our study found that there is a relationship between thyroid hormone and all-cause readmission or death risk in HF patients with stroke. Thyroid hormone is critical for the development and function of nearly all organs and tissues, with the cardiovascular system being one of the major targets. Thyroid hormone is known to increase the heart rate, increase cardiac contractility, alter systolic and diastolic function, and decrease systematic vascular resistance [16]. Thyroid dysfunction, even in the subclinical range, is associated with increased incidences of cardiovascular risk factors and disease [16]. This can be explained by the positive inotropic, chronotropic, and lusitropic effects of tri-iodothyronine [17,18]. Thyroid hormone levels are often altered in patients with a history of stroke: approximately 28% of ischemic stroke patients have TSH concentrations outside the reference range [19]. A complex relationship has been described between thyroid function and recovery from ischemic stroke, probably because thyroid hormone has both neurotoxic and neuroprotective effects [20,21]. Ischemic stroke may change the secretion of TSH in the brain, and its specific pathogenesis is still not clearly defined. Thus, the thyroid function should be monitored closely in patients with HF.

### 4.3. DBP

Our study found that when DBP is lower than 60 mmHg, there was an increased risk of 3-month all-cause readmission or death. This result might partly be explained by the reduction in coronary perfusion during the diastole of the heart, which could increase the risks of coronary and cardiovascular events in HF patients. A low DBP was also associated with subclinical myocardial damage and cardiovascular events [22,23,24]. Another possible explanation for this may be that ischemic stroke survivors with low DBP suffer from poor cerebral perfusion, which may lead to worse outcomes. There is evidence that significantly lowering blood pressure reduces cardiovascular events, although it has also been shown that an intensive strategy does increase the risks of some adverse effects. Additionally, a previous study preformed in China found a U-shaped relationship between DBP and nonfatal strokes in patients after stroke [25]. Another study also indicated that a DBP lower than 75 mmHg was associated with an increased risk of all-cause mortality in patients with a history of ischemic stroke [26]. In the present study, the enrolled patients had low DBPs so that we could only see part of the U-shaped curve, which is consistent with the research of Zhao M et al. [25].

### 4.4. Beta-Blockers 

We observed that stroke-associated increased risk of death or readmission was higher in those not receiving beta-blockers no matter the timeframe, i.e., 3 months or 1 year. The use of beta-blockers may also have a neuroprotective effect in patients with ischemic stroke by reducing the increased sympathetic activity, which in turn may improve prognosis [27]. An autonomic shift with increased sympathetic activity has been repeatedly observed in acute stroke patients and is related to worse outcomes [28,29,30]. The possible pathophysiological mechanisms behind this include increased cardiovascular complications, arrhythmias, blood pressure derangements, nondiabetic hyperglycemia, and the promotion of secondary brain injury because of local inflammation and edema [31]. Thus, the modification of impaired autonomic functions may have important therapeutic implications in acute ischemic stroke patients. Our results add to the evidence that exposure to beta-blockers is associated with improved survival in HF patients, particularly in HF patients with prior ischemic stroke.

### 4.5. Hypoalbuminemia and Hyperglycosemia

Similarly, to prior reports, we found that hypoalbuminemia is associated with unfavorable outcomes in HF. Hypoalbuminemia is common in patients with HF, especially in the elderly, likely in relation to their higher rate of frailty [32]. In HF, hypoalbuminemia may be a marker of comorbidity burden, inflammatory state, malnutrition, and cachexia [33]. Low serum albumin levels are associated with increased risks of HF onset and progression. Indeed, hypoalbuminemia may promote pulmonary congestion; myocardial edema and subsequent worsening of myocardial dysfunction, diuretic resistance, and fluid retention; and decreases in antioxidant functions and anti-inflammatory properties [34,35]. Additionally, the risk factors for patients with ischemic stroke are modifiable and can be influenced by appropriate therapy or lifestyle changes, which may exacerbate the nutritional condition [36,37]. Thus, nutritional complications frequently occur among patients undergoing HF and ischemic stroke, and the importance of nutrition in these patients should be emphasized. About 1/3 of patients with HF also have diabetes malleus (DM). In this study, the mean HbA1c % level was high in both groups. Hyperglycemia increases the risk of diastolic or/and systolic left ventricular dysfunction. Appropriate HF treatment, combined with good glycemic control, seems to offer the best chances for beneficial outcomes [38].

## 5. Limitations

There are a few limitations to this study. Firstly, the conclusions of this study are based on only observational study and not a randomized trial; no intervention was implemented. The modest number of events observed made statistical modeling prone to overfitting and limited the analysis of individual events and the ability to control for potential confounders. Due to the influence of the sample size, only part of the curve could be observed for variables that might have had a U-shaped relationship with readmission risk. If we have a large enough sample size, we can observe complete readmission risk curves for some variables. Secondly, this study did not assess the impacts of patient-reported outcomes, such as functional status and quality of life, on the risk of readmission. 

## 6. Conclusions

Without β-blockers, lower serum albumin and abnormal thyroid function increase readmission and death risks in elderly HF patients who have had an ischemic stroke at 3 months and 1 year after discharge. The other factors, such as being without ACEI/ARB and a high heart rate, only increase risks at 3 months or 1 year, not both. These risk factors should be routinely assessed in HF patients with a history of ischemic stroke.

## Figures and Tables

**Table 1:** Baseline characteristics of patients with HF by a history of ischemic stroke.

Variable	Total Cohort		
	No Prior Ischemic Stroke (*n* = 611)	Prior Ischemic Stroke (*n* = 340)	*p* Value
Age, years	76.1 ± 6.4	78.6 ± 6.5	0.680
Man	313 (51.2)	192 (56.5)	0.120
SBP, mmHg	130.1 ± 24.0	130.2 ± 26.1	0.880
DBP, mmHg	73.3 ± 12.7	74.1 ± 15.4	0.041
Heart rate, bpm	78.6 ± 17.2	77.5 ± 15.8	0.331
BMI, kg/m^2^	24.3 ± 4.2	24.8 ± 3.8	0.790
History of smoking	246 (40.9)	136 (30.5)	0.892
Co-morbidities			
Coronary heart disease	378 (61.9)	258 (75.9)	<0.001
Hypertension	453 (74.1)	287 (84.4)	<0.001
Dyslipidemia	296 (48.4)	187 (55.0)	0.052
Diabetes mellitus	225 (36.8)	147 (43.2)	0.053
Atrial fibrillation	195 (31.9)	128 (37.6)	0.075
Anemia	213 (34.9)	135 (39.7)	0.138
CKD	71 (11.6)	75 (22.1)	<0.001
Well control Cancer	125 (20.5)	85 (25.0)	0.108
COPD	111 (18.2)	45 (13.2)	0.046
Thyroid dysfunction	33 (5.5)	27 (7.9)	0.145
Laboratory findings			
White cell, 10^9^/L *	7.6 ± 3.4	7.6 ± 3.2	0.773
Neutrophil ratio, % *	70.3 ± 12.5	70.7 ± 11.2	0.017
Hemoglobin, g/L	118.2 ± 22.4	117.9 ± 20.6	0.164
Serum albumin, g/L *	36.6 ± 4.6	36.5 ± 4.8	0.197
Glucose, mmol/L	7.3 ± 3.3	7.7 ± 3.6	0.805
HbA1c, %	6.8 ± 1.4	6.6 ± 1.2	0.359
Serum sodium, mmol/L *	139.8 ± 4.5	138.7 ± 5.3	0.110
Serum potassium, mmol/L *	4.1 ± 0.6	4.2 ± 0.6	0.044
Serum calcium, mmol/L	2.1 ± 0.3	2.1 ± 0.3	0.988
Total cholesterol, mmol/L	3.9 ± 1.0	3.9 ± 1.1	0.056
Triglycerides, mmol/L	1.2 ± 0.8	1.3 ± 1.0	0.191
LDL-C, mmol/L	2.3 ± 0.8	2.4 ± 1.0	0.028
HDL-C, mmol/L	1.0 ± 0.3	0.9 ± 0.3	0.131
Total bilirubin	15.4 ± 12.3	14.8 ± 9.6	0.785
Direct bilirubin	5.8 ± 8.2	5.4 ± 5.5	0.682
Serum creatinine, μmol/L	110.3 ± 104.7	107.3 ± 80.8	0.049
eGFR, ml/min/1.73 m^2^	68.7 ± 26.3	64.5 ± 25.8	0.790
≥60	380 (65.4)	182 (57.8)	0.025
<60	201 (34.6)	133 (42.2)	
≥30	553 (90.5)	308 (90.6)	0.967
<30	58 (9.5)	32 (9.4)	
CRP *	46.0 ± 65.0	48.0 ± 56.6	0.955
NT-proBNP, pg/mL *	1946.0 (734.1, 5284.1)	1495.0 (466.5, 3477.75)	0.003
TSH, μIU/mL *	4.0 ± 12.2	3.0 ± 5.3	0.046
T3, ng/dL *	83.6 ± 38.1	82.1 ± 25.0	0.204
T4, ug/dL	7.9 ± 2.3	7.7 ± 2.1	0.724
FT3, pmol/L *	3.7 ± 1.1	3.7 ± 0.7	0.039
FT4, pmol/L *	7.9 ± 2.3	7.7 ± 2.1	0.329
LVEF, %	56.7± 13.2	60.2 ± 11.6	<0.001
HFrEF	58 (13.3)	15 (7.7)	
HfmrEF	66 (15.2)	24 (12.4)	0.018
HfpEF	311 (71.5)	155 (79.9)	
Medications at discharge			
ACEIs/ARBs/ARNI	354 (57.9)	210 (61.8)	0.249
β-blockers	365 (59.7)	202 (59.4)	0.922
MRAs	233 (38.1)	111 (32.6)	0.090
Statins	265 (43.4)	151 (44.4)	0.757
Loop diuretics	440 (72.0)	283 (83.2)	<0.001
Nitrates	274 (44.8)	185 (54.4)	0.005
Digoxin	145 (23.7)	62 (18.2)	0.047
Antiplatelet drugs	284 (46.5)	202 (59.4)	<0.001
Anticoagulants	128 (20.9)	38 (11.2)	<0.001
Calcium channel blocker	155 (25.4)	122 (35.9)	0.001
Length of stay, days *	19.9± 18.0	22.0 ± 18.6	0.733

Values are mean ± standard deviation or number (%), unless indicated otherwise. * Median (interquartile range). BMI, body mass index; SBP, systolic blood pressure; DBP, diastolic blood pressure; CKD, chronic kidney disease; COPD, chronic obstructive pulmonary disease; NT-proBNP, N-terminal pro-B-type natriuretic peptide; eGFR, estimated glomerular filtration rate; LVEF, left ventricular ejection fraction; LDL-C, low-density lipoprotein cholesterol; HDL-C, high-density lipoprotein cholesterol; CRP, C-reactive protein; Hs-CRP, high-sensitivity C-reactive protein; TSH, thyroid stimulating hormone; T3, triiodothyronine; T4, thyroxin; FT3, free triiodothyronine; FT4, free thyroxin; ACE, angiotensin-converting enzyme; ARB, angiotensin receptor blocker; ARNI, angiotensin receptor-neprilysin inhibitor; MRAs, mineralocorticoid receptor antagonists.

**Table 2:** Clinical outcomes in the total cohort.

	Events (%)		HR (95% CI)	*p* Value
	No Prior Ischemic Stroke (*n* = 611)	Prior Ischemic Stroke (*n* = 340)		
3-month outcomes				
All-cause readmission	97 (15.8)	113 (33.2)	2.33 (1.76–3.06)	<0.001
HF readmission	56 (9.2)	22 (6.5)	0.80 (0.49–1.31)	0.378
Non-HF readmission	40 (6.5)	91 (26.8)	4.48 (3.09–6.51)	<0.001
All-cause death	70 (11.5)	32 (9.4)	0.82 (0.54–1.25)	0.363
All-cause death or readmission	199 (32.6)	141 (41.5)	1.75 (1.39–2.19)	<0.001
1-year outcomes				
All-cause readmission	175 (32.2)	181 (58.4)	2.27 (1.84–2.79)	<0.001
HF readmission	102 (16.7)	42 (12.4)	0.92 (0.64–1.32)	0.653
Non-HF readmission	73 (13.4)	139 (44.8)	4.12 (3.10–5.47)	<0.001
All-cause death	73 (11.9)	35 (10.3)	0.80 (0.53–1.20)	0.797
All-cause death or readmission	243 (39.8)	211 (62.1)	1.87 (1.55–2.25)	<0.001

HF indicates heart failure; HR, hazard ratios; CI, confidence interval.

**Table 3:** Patient characteristics and multivariate analysis of factors for 3-month all-cause readmission or death in elderly HF patients with a history of ischemic stroke.

	**Univariate Anslysis**				**Multivariate Analysis**	
**Variable**	**without 3-Month Readmission or Death** ***n* = 199 (58.5)**	**with 3-Month** **Readmission or Death** ***n* = 141 (41.5)**	**HR (95%CI)**	***p* Value**	**HR (95%CI)**	***p* Value**
Age, years	77.51 ± 6.11	80.06 ± 6.80	1.09 (0.91–1.30)	0.220		
Man, *n* (%)	116 (58.3)	76 (53.9)	1.61 (0.84–3.05)	0.421		
SBP, mmHg	133.35 ± 20.48	125.67 ± 32.06	1.49 (1.11–2.00)	0.006	1.00 (0.99–1.01)	0.447
DBP, mmHg	75.05 ± 12.49	72.87 ± 18.65	0.96 (0.94–0.98)	0.026	0.99 (0.97–1.00)	0.045
Length of stay, days	19.01 ± 15.58	26.16 ± 21.48	1.00 (0.99–1.01)	<0.001	1.00 (0.99–1.01)	0.837
eGFR, mL/min/1.73 m^2^	66.57 ± 23.83	61.00 ± 28.61	1.55 (1.28–1.87)	0.006	1.00 (0.99–1.01)	0.742
eGFR ≥ 60	120 (61.2)	62 (52.1)				
Infection	22 (11.1)	31 (22.3)	1.87 (1.68–2.08)	0.006	1.68 (1.00–2.82)	0.050
Serum albumin, g/L	37.78 ± 4.27	34.69 ± 5.00	0.90 (0.87–0.93)	0.018	0.95 (0.90–0.99)	0.025
Serum sodium, mmol/L	139.47 ± 4.31	137.71 ± 6.37	0.95 (0.93–0.97)	0.010	0.98 (0.95–1.02)	0.336
Serum potassium, mmol/L	4.08 ± 0.54	4.28 ± 0.65	1.14 (0.81–1.60)	0.068	1.08 (0.76–1.54)	0.676
Serum creatinine, μmol/L	97.44 ± 53.31	121.39 ± 107.26	1.45 (1.24–1.70)	<0.001	1.16(0.87–1.55)	0.341
TSH, μIU/mL	2.61 ± 3.17	3.60 ± 7.68	1.06 (0.99–1.13)	0.033	1.04 (1.01–1.08)	0.017
Coronary heart disease	139 (70.2)	112 (79.4)	0.84 (0.71–0.99)	0.041	0.87 (0.51–1.46)	0.588
Cardiomyopathy	1 (0.5)	9 (6.4)	4.10 (1.30–12.9)	0.001	3.84 (1.52–9.72)	0.005
Valvular disorders	14 (7.4)	1 (0.7)	0.25 (0.08–0.78)	0.001	0.13 (0.02–1.01)	0.051
Anemia	67 (33.7)	68 (48.2)	0.86 (0.78–0.94)	0.007	0.69 (0.43–1.09)	0.113
Cancer	43 (21.6)	42 (29.8)	0.80 (0.59–1.09)	0.088	0.78 (0.50–1.23)	0.290
COPD	20 (10.1)	25 (17.7)	0.78 (0.64–0.94)	0.041	0.85 (0.48–1.50)	0.566
ACE-inhibitors/ARBs/ARNI	150 (75.4)	60 (42.6)	0.59 (0.37–0.93)	<0.001	0.59 (0.37–0.93)	0.024
β-Blockers	142 (71.4)	60 (42.6)	0.68 (0.47–0.97)	<0.001	0.62 (0.40–0.95)	0.029
MRAs	76 (38.2)	35 (24.8)	0.88 (0.80–0.97)	0.009	0.85 (0.53–1.38)	0.513
Antiplatelet drugs	148 (74.4)	37 (26.2)	0.51 (0.32–0.82)	<0.001	0.51 (0.32–0.82)	0.005
Statins	114 (57.3)	37 (26.2)	0.69 (0.41–1.16)	<0.001	0.69 (0.41–1.16)	0.159

SBP, systolic blood pressure; DBP, diastolic blood pressure; COPD, chronic obstructive pulmonary disease; NT-proBNP, N-terminal pro-B-type natriuretic peptide; eGFR, estimated glomerular filtration rate; LVEF, left ventricular ejection fraction; TSH, thyroid stimulating hormone; ACE, angiotensin-converting enzyme; ARB, angiotensin receptor blocker; ARNI, angiotensin receptor-neprilysin inhibitor; MRAs, mineralocorticoid receptor antagonists.

**Table 4:** Patient characteristics and multivariate analysis of factors for 1-year all-cause readmission or death in elderly HF patients with prior ischemic stroke.

	Univariate Analysis				Multivariate Analysis	
Variable	without 1 Year Readmission Or Death *n* = 129 (37.9)	with 1 Year Readmission Or Death *n* = 211 (62.1)	HR (95%CI)	*p* Value	HR (95%CI)	*p* Value
Age, years	76.53 ± 5.75	79.82 ± 6.66	1.34(0.91–1.97)	0.147		
Man, *n* (%)	76 (58.9)	116 (55.0)	1.85(0.87–3.93)	0.477		
SBP, mmHg	133.44 ± 20.50	128.17 ± 28.93	1.37(0.97–1.09)	0.086	1.00 (0.99–1.01)	0.799
DBP, mmHg	75.30 ± 11.53	73.43 ± 17.28	0.87 (0.78–0.97)	0.010	0.99 (0.98–1.00)	0.164
Heart rate, bpm	78.29 ± 18.08	77.09 ± 14.18	0.89 (0.76–1.04)	0.058	0.99 (0.97–1.00)	0.040
Length of stay, days	17.91 ± 10.86	24.45 ± 21.64	1.17 (0.97–1.41)	0.010	1.00 (0.99–1.01)	0.684
Hemoglobin, g/L	122.23 ± 18.53	115.25 ± 21.32	1.17 (1.08–1.26)	0.034	1.00 (0.99–1.02)	0.401
eGFR, mL/min/1.73 m^2^	68.08 ± 24.72	61.96 ± 26.35	1.03 (0.98–1.08)	0.124		
eGFR ≥ 60	82 (63.6)	100 (53.8)	0.97 (0.64–1.47)	0.082	0.97 (0.64–1.47)	0.883
Infection	20 (15.5)	33 (15.8)	2.51 (1.48–4.26)	0.944		
Serum albumin, g/L	37.91 ± 4.18	35.64 ± 5.00	0.89 (0.81–0.97)	0.013	0.93 (0.87–0.97)	0.003
Serum potassium, mmol/L	4.06 ± 0.54	4.22 ± 0.62	1.55 (0.94–2.55)	0.095	1.41 (0.98–2.03)	0.063
CRP	22.63 ± 28.83	59.35 ± 62.25	1.35 (1.12–1.62)	0.001	1.01 (1.00–1.02)	0.028
TSH, μIU/mL	2.79 ± 3.71	3.10 ± 6.13	1.45 (0.872–2.42)	0.442		
FT4, pmol/L	15.77 ± 2.77	16.25 ± 3.54	1.15 (1.09–1.21)	0.034	1.08 (1.01–1.15)	0.018
COPD	11 (8.5)	34 (16.1)	0.92 (0.86–0.98)	0.040	0.88 (0.44–1.77)	0.727
ACE-inhibitors/ARBs/ARNI	105(81.4)	105 (49.8)	0.81 (0.69–0.94)	<0.001	0.68 (0.44–1.04)	0.075
β-Blockers	102 (79.1)	100 (47.4)	0.61 (0.41–0.91)	<0.001	0.53 (0.35–0.80)	0.003
MRAs	51 (39.5)	60 (28.4)	0.97 (0.67–1.40)	0.035	1.14 (0.74–1.76)	0.543
Antiplatelet drugs	96 (74.4)	106 (50.2)	0.58 (0.437–0.92)	<0.001	0.76 (0.48–1.19)	0.232
Statins	79 (61.2)	72 (34.1)	0.62 (0.42–0.91)	<0.001	0.73 (0.47–1.16)	0.182

SBP, systolic blood pressure; DBP, diastolic blood pressure; COPD, chronic obstructive pulmonary disease; NT-proBNP, N-terminal pro-B-type natriuretic peptide; eGFR, estimated glomerular filtration rate; LVEF, left ventricular ejection fraction; CRP, C-reactive protein; TSH, thyroid stimulating hormone; FT4, free thyroxin; ACE, angiotensin-converting enzyme; ARB, angiotensin receptor blocker; ARNI, angiotensin receptor-neprilysin inhibitor; MRAs, mineralocorticoid receptor antagonists.

## Data Availability

The data that support the findings of this study are available from Xue-Bin Li M.D.
